# Hyperchloremia in critically ill patients: association with outcomes and prediction using electronic health record data

**DOI:** 10.1186/s12911-020-01326-4

**Published:** 2020-12-15

**Authors:** Pete Yeh, Yiheng Pan, L. Nelson Sanchez-Pinto, Yuan Luo

**Affiliations:** 1 Feinberg School of Medicine, Northwestern University, Chicago, IL USA; 2 Department of Electrical Engineering and Computer Science, Northwestern University, Evanston, IL USA; 3 Department of Pediatrics (Critical Care), Feinberg School of Medicine, Northwestern University, Chicago, IL USA; 4 Department of Preventive Medicine (Health and Biomedical Informatics), Feinberg School of Medicine, Northwestern University, Chicago, IL USA

**Keywords:** Biomedical informatics, Decision support systems, Machine learning, Predictive models

## Abstract

**Background:**

Increased chloride in the context of intravenous fluid chloride load and serum chloride levels (hyperchloremia) have previously been associated with increased morbidity and mortality in select subpopulations of intensive care unit (ICU) patients (e.g patients with sepsis). Here, we study the general ICU population of the Medical Information Mart for Intensive Care III (MIMIC-III) database to corroborate these associations, and propose a supervised learning model for the prediction of hyperchloremia in ICU patients.

**Methods:**

We assessed hyperchloremia and chloride load and their associations with several outcomes (ICU mortality, new acute kidney injury [AKI] by day 7, and multiple organ dysfunction syndrome [MODS] on day 7) using regression analysis. Four predictive supervised learning classifiers were trained to predict hyperchloremia using features representative of clinical records from the first 24h of adult ICU stays.

**Results:**

Hyperchloremia was shown to have an independent association with increased odds of ICU mortality, new AKI by day 7, and MODS on day 7. High chloride load was also associated with increased odds of ICU mortality. Our best performing supervised learning model predicted second-day hyperchloremia with an AUC of 0.76 and a number needed to alert (NNA) of 7—a clinically-actionable rate.

**Conclusions:**

Our results support the use of predictive models to aid clinicians in monitoring for and preventing hyperchloremia in high-risk patients and offers an opportunity to improve patient outcomes.

## Background

Intravenous (IV) fluids are commonplace in the critical care setting for good reason—they are low-risk, go-to interventions for patients with fluid deficits and electrolyte imbalances. Recent studies have reexamined the effects of these fluids, however, and mounting evidence cautions that aggressive doses that are still within reference therapeutic ranges may lead to adverse outcomes ranging from organ damage to in-hospital mortality [1]. Particular concern has been raised regarding chloride, an oft-unnoticed component of many standard IV fluids such as normal saline. Higher rates of in-hospital mortality were observed with elevated IV fluid chloride content during resuscitation with large fluid volumes [2] as well as in patients with sepsis [3]. Additionally, hyperchloremia in patients with sepsis has been linked to higher rates of acute kidney injury (AKI) [4] and mortality [5]. Conversely, low-chloride strategies demonstrated reductions in AKI and renal replacement therapy [6]. These findings warrant further investigation into the merits of shifting from the traditional approach of chloride-liberal fluid administration to a chloride-restrictive one, which could be of benefit to critically ill patients.

Electronic health records (EHRs) collect and store countless data points for each intensive care unit (ICU) patient [7] and contain a wealth of information on demographics, medical interventions, measurements, outcomes, and more. By mining EHR data from the general ICU population of the Medical Information Mart for Intensive Care III (MIMIC-III) [8], we retrospectively studied hyperchloremia and high chloride load in IV fluids and evaluated their associations with patient mortality and organ dysfunction. As there have been many promising developments in clinical event prediction using machine learning, we also propose a predictive model for hyperchloremia using this EHR data. This model can alert clinicians to patients at high risk for hyperchloremia and provide opportunities for improved chloride management, which may in turn improve patient outcomes.

MIMIC-III is a well-studied dataset for good reason—it contains a sizeable ICU population of over 40,000 patients and spans over 10 years of data from 2001 to 2012. As such, numerous predictive models have been built on specific subgroups of interest, such as patients with kidney injury [9], pneumonia [10], myocardial infarction [11], sepsis [12], and more. Existing models typically predict outcomes such as mortality [9, 13–15], ICU readmission [16–18], AKI [19–21], and other complications [22], with varying AUCs ranging from 0.65 to 0.9.

In light of the specific focus of these studies, there still exists a knowledge gap for predictive modeling in general ICU populations, especially modeling focused on treatment management. A focus on treatment management is important for clinical decision-making as outcome-focused predictions may be of limited clinical utility despite high prognostic value. Mortality, for example, can result from any number of potential factors, and a prediction that mortality is likely to occur is difficult to act upon without a clear contributing cause.

Our study thus aims to expand on existing research by analyzing hyperchloremia and its associations with several key outcomes in the general adult ICU population of MIMIC-III and then predicting hyperchloremia for this population. Chloride administration in the ICU is actively managed via IV fluids, and thus these predictions can prompt immediate interventions to reduce chloride load and limit hyperchloremia. If chloride load and hyperchloremia are indeed causally linked to poor outcomes, this framework has potential for improving patient care.

This manuscript is an extension of our previously published work on predicting hyperchloremia [23]. Here, we extend our analysis to further evaluate associations between chloride load and patient outcomes, assess the impact of individual features, and examine the implications of false positive and false negative predictions. Additionally, the methods and results sections have been extended to elaborate on nuances in data preprocessing, feature selection, and hyperparameter tuning.

## Methods

### Statistical analysis

With a focus on the first 7 days of critical illness, we evaluated the relationship between chloride and patient outcomes in the ICU using retrospective data extracted from MIMIC-III. In particular, we evaluated the associations between chloride load and outcomes as well as the associations between hyperchloremia and outcomes.

Chloride load was represented as the average daily chloride input given to a patient. Hyperchloremia was represented as a binary variable—whether or not hyperchloremia occurred in the first 7 days, which we defined as any serum chloride measurement of 110 mEq/L or greater. We also represented hyperchloremia as two quantitative variables: the number of days in which hyperchloremia occurred and the maximum serum chloride measured over the first 7 days.

A seven-day time span provides a sufficiently large window in which measurable adverse outcomes can develop. We used several objective measures of morbidity and mortality:

- Mortality during the ICU stay (ICU mortality)
- New AKI by day 7
- Multiple Organ Dysfunction Syndrome (MODS) [24] on day 7

MODS was considered positive if two or more Sequential Organ Failure Assessment (SOFA) [25] sub-scores were 2 or greater. New AKI was considered positive if AKI stage [26] ever increased (i.e. worsened) from baseline within the first 7 days. In other words, a patient who presented to the ICU with stage 3 AKI (the highest possible stage) would not be considered to have new AKI, whereas a patient that presents with stage 2 AKI and progresses to stage 3 would be considered to have new AKI.

**Fig. 1:**
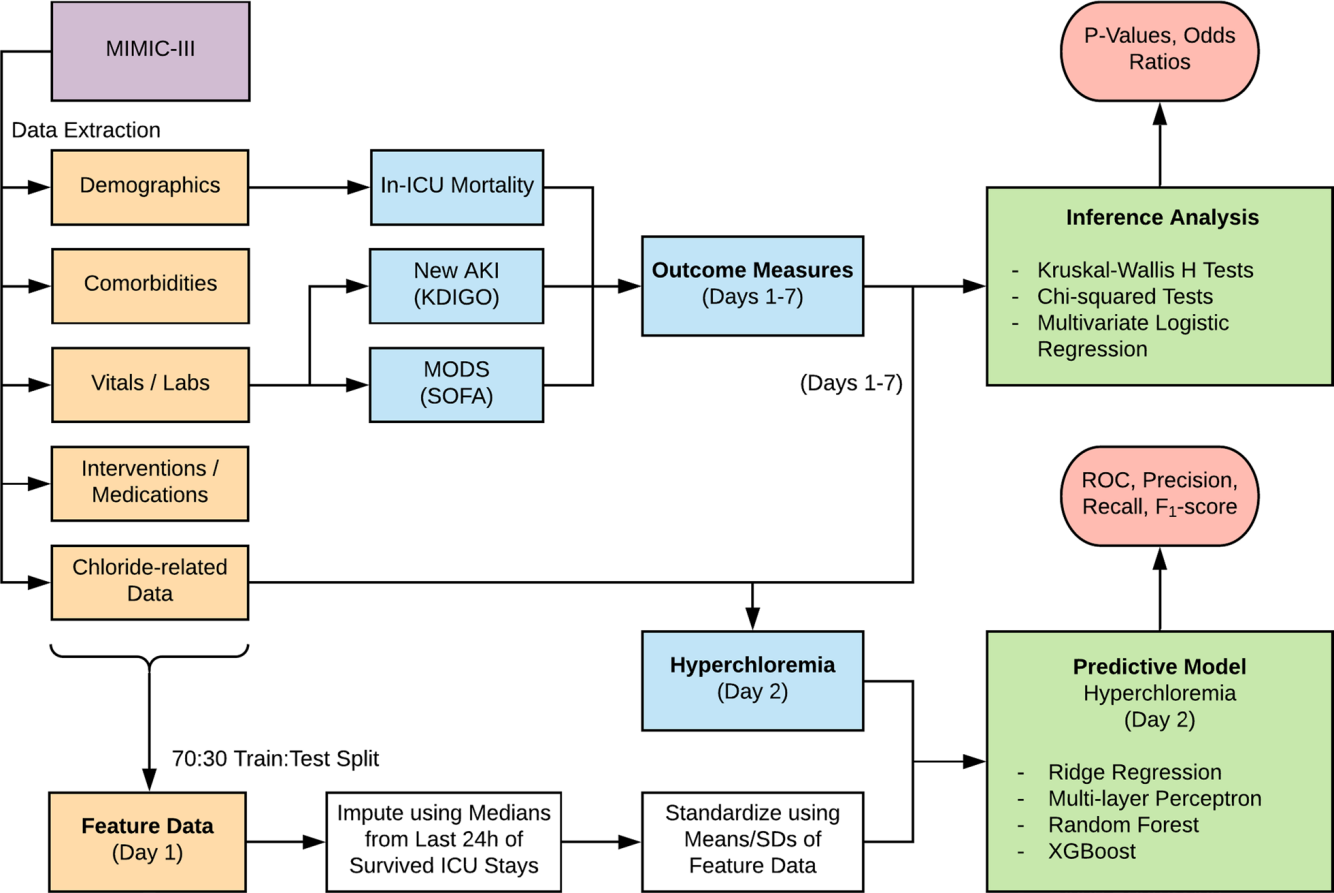
Study design. Data was extracted from MIMIC-III and organized into feature sets and outcome measures based on clinical guidelines and expertise. Statistical analysis determined the significance of associations. Imputation and standardization of both training and testing set data were performed using only training set medians and means

We utilized Kruskal-Wallis H tests and chi-squared tests to assess differences in chloride status between patients who were negative for each outcome and patients who were positive for them. We also modeled chloride-outcome associations using multivariate logistic regression to control for demographics and severity of illness on admission (Fig. 1).

### Prediction modeling

#### Feature selection

Using feature data aggregated from the initial 24 h (“day 1”) of patient ICU stays, we trained supervised learning models to predict the likelihood that hyperchloremia would occur in the following 24 h (“day 2”). Our feature set included chloride-related data (net fluid balance, total chloride load, maximum serum chloride), comorbidities on admission, demographics, interventions, laboratory test results, medications, and vitals. Chloride load included any fluid with chloride (e.g. normal saline, potassium chloride, etc.), converted into milliequivalent (mEq) quantities using standard ratios. Cutoffs were chosen based on clinical intuition to exclude nonsensical values (e.g. serum chloride $\ge$ 160 mEq/L, daily chloride input $\ge$ 5000 mEq, net fluid balance of $\ge$ 30,000 mL, negative values, out-of-order start/end times). We identified comorbidities of interest using the Elixhauser Index [27] and ICD-9 codes. Of note, comorbidities are not timestamped in MIMIC-III and are instead only tied to the hospital admissions in which they were recorded, and thus comorbidity features were limited to those assigned in *prior* hospital admissions—comorbidities already known at the time of the current admission.

These variables were chosen based on clinical expertise and prior literature and were only included if statistical significance could be demonstrated using two-sample t-tests and chi-squared tests. 34 features were ultimately fed into our models, each with statistically significant differences (*p* value < 0.05) between hyperchloremic and non-hyperchloremic patients on day 2. We standardized non-binary variables by subtracting means and scaling to unit variance. Only training set data was used to determine statistical significance for feature selection, and only training set data was used to calculate means and standard deviations for feature standardization.

Our study focuses on initial adult ICU admissions based on the expectation that pediatric patients and ICU re-admissions exhibit uniquely different physiologic behaviors. Thus, ICU stays were excluded if the patient was below 18 years of age or in the ICU for a readmission within a hospitalization. Of the remaining 49,696 ICU stays, we further excluded 16,366 (32.9%) records as these patients were either already hyperchloremic or did not have chloride data on day 1 (we did not impute serum chloride measurements on day 1). The resulting 33,330 rows of unique ICU stays were then divided on a 70:30 train:test split and the testing set was held out for performance evaluation.

#### Imputing missing data

EHRs inherently tend to lack records for events that do not occur. For example, a patient that did not receive chloride would have no record of chloride administration, and vice versa. Thus, for features that would not be present at a “healthy” baseline—interventions (chloride administration, fluid input/output), medications, and comorbidities on admission—a zero value was inferred in the absence of data.

For imputation of measurements that are non-zero at a “healthy” baseline—vitals, laboratory tests—we determined the median of each feature using training set records limited to the 24 h prior to ICU discharges (i.e. the calculation did not include patients who did not survive). The final 24 h in the ICU of patients who survive are generally representative of a “healthier” state compared to earlier stages in the ICU.

Patients with no records of serum chloride measurements throughout day 2 were presumed non-hyperchloremic for that day. Of note, the vast majority of such patients also had no recorded chloride measurements beyond day 2—we assumed that this would not be the case had their clinicians been concerned for hyperchloremia.

#### Machine learning classifiers

We evaluated four classifiers: ridge regression, random forest, XGBoost [28], and multi-layer perceptron. Each classifier predicts probabilities of hyperchloremia ($\ge$ 110 mEq/L) occurring on day 2 for each patient. These probabilities were then converted into binary classifications using thresholds that maximized the Youden’s J statistic of training set predictions.

The low prevalence of day 2 hyperchloremia in our dataset (Table 1) necessitated additional compensatory steps. We configured the ridge regression and XGBoost classifiers to assign weights based on prevalence. For the random forest and multi-layer perceptron classifiers, we chose to down-sample our training set by removing, at random, 90% of patients who did not develop hyperchloremia on day 2. This resulted in a final training set size of 3,560 rows for these classifiers with a prevalence of 38.29%, which was sufficiently large and balanced.

**Table 1:** Prevalence of hyperchloremia on day 2 by dataset

Dataset	Hyperchloremic	Non-hyperchloremic	Total	Prevalence (%)
Training	1363	21,968	23,331	5.84
Testing	629	9370	9999	6.29
Whole	1992	31,338	33,330	5.98

#### Performance evaluation

Classifier performance was represented via precision, recall, F_1_-scores, and receiver operating characteristic (ROC) curves (Fig. 1). We also plotted precision-recall curves to illustrate the trade-offs that can be made between precision and recall. These metrics are sensitive to imbalanced outcomes, which is important for our use case as the prevalence of hyperchloremia is low.

#### Feature analysis

We inspected the coefficients of our fitted regression model to identify features that were most predictive of and/or protective against hyperchloremia. Comparing the relative magnitudes allows us to corroborate our baseline understanding of features that we expect to be significant, and perhaps more importantly it also highlights features that are *unexpectedly* significant. This could, in turn, yield new clinical insight into associations that are important for clinical consideration.

#### Error analysis

We also analyzed patient records for several incorrect predictions to identify characteristics that are prone to misclassification. Insight into commonalities within the false positive cohort can help us better understand the assumptions and limitations of our models.

## Results

### Statistical analysis

Table 2 lists characteristics and outcomes pertinent to our study population.

**Table 2:** Clinical characteristics and outcomes by occurrence of hyperchloremia in days 1–7

	Hyperchloremic	Non-hyperchloremic	All patients	
	(*n* = 18181)	(*n* = 29893)	(*n* = 48074)	*p* value
***Demographics***				
Age, median (IQR)	66.9 (53.8–78.5)	65.0 (52.4–77.4)	65.7 (52.9–77.8)	< 0.001
Female, *n* (%)	8324 (45.8)	12684 (42.4)	21008 (43.7)	< 0.001
***Race***, ***n*** (%)				
Asian	518 (2.8)	603 (2.0)	1121 (2.3)	< 0.001
Black	1614 (8.9)	3006 (10.1)	4620 (9.6)	
Hispanic	598 (3.3)	1053 (3.5)	1651 (3.4)	
White	12930 (71.1)	21477 (71.8)	34407 (71.6)	
Other (unreported)	2521 (13.9)	3754 (12.6)	6275 (13.1)	
***Comorbid conditions***, ***n*** (%)				
Cancer	1488 (8.2)	3088 (10.3)	4576 (9.5)	< 0.001
Cardiovascular	9838 (54.1)	16385 (54.8)	26223 (54.5)	0.137
Diabetes	4625 (25.4)	8436 (28.2)	13061 (27.2)	< 0.001
Hepatic	1989 (10.9)	2960 (9.9)	4949 (10.3)	< 0.001
Renal	2589 (14.2)	5279 (17.7)	7868 (16.4)	< 0.001
Respiratory	3434 (18.9)	7263 (24.3)	10697 (22.2)	< 0.001
Multiple comorbidities	15304 (84.2)	24819 (83.0)	40123 (83.5)	0.001
***Outcome***, *n* (%)				
ICU mortality	1855 (10.2)	1703 (5.7)	3558 (7.4)	< 0.001
New AKI by day 7	7788 (42.8)	9445 (31.6)	17233 (35.8)	< 0.001
MODS on day 7	1109 (6.1)	781 (2.6)	1890 (3.9)	< 0.001

Univariate statistical analysis demonstrated that increased maximum serum chloride level, hyperchloremia ($\ge 110$ mEq/L), increased number of days in which hyperchloremia occurred, and increased chloride load in IV fluids each demonstrated increases in ICU mortality, new AKI by day 7, and MODS on day 7 that had statistical significance (*p* value $< 0.001$).

Multivariate regression analysis results are presented in Table 3. Odds ratios for the outcomes of interest were determined after adjusting for potentially confounding demographic variables (age, gender, ethnicity) and severity of illness on admission (represented using the SOFA score). As detailed in Table 3, all three measures of hyperchloremia were independently associated with increased ICU mortality, new AKI by day 7, and MODS on day 7. Chloride load was only independently associated with increased ICU mortality and had an inverse relationship with MODS on day 7.

**Table 3:** Adjusted odds ratios for chloride-outcome associations

	Adjusted odds ratios [95% confidence interval]		
	ICU Mortality	New AKI by day 7	MODS on day 7
*Over days 1–7*			
Max. serum chloride (per mEq/L)	1.035 [1.029, 1.040]	1.049 [1.046, 1.053]	1.056 [1.048, 1.063]
Hyperchloremia	1.376 [1.280, 1.481]	1.680 [1.615, 1.747]	1.823 [1.652, 2.012]
Hyperchloremic days	1.186 [1.161, 1.211]	1.154 [1.137, 1.170]	1.402 [1.369, 1.436]
Avg. daily chloride load (per 100 mEq)	1.144 [1.122, 1.165]	0.995 [0.983, 1.007]	0.920 [0.894, 0.946]

### Prediction modeling

#### Selected features

All chloride-related features that we initially selected were included by default. The following features were also included as they showed statistical significance:

- Demographics: Age, Ethnicity, Gender
- Comorbidities:
  - Cardiovascular (Congestive Heart Failure, Hypertension, Pulmonary Circulation Disease)
  - Chronic Obstructive Pulmonary Disease
  - Complicated Diabetes, Uncomplicated Diabetes
  - Renal Failure
  - Alcohol Abuse, Depression
  - Paralysis
- Vitals:
  - Max. Heart Rate, Min. Systolic Blood Pressure, Min. Diastolic Blood Pressure
  - Max. Respiratory Rate, Min. SpO_2_
  - Min. Glasgow Coma Score
  - Weight, Max. Temperature ($^{\circ }$C)
- Laboratory measurements:
  - Max. Sodium, Potassium, International Normalized Ratio (INR)
  - Min. Potassium, Bicarbonate, Total Calcium
- Interventions:
  - Norepinephrine
  - Airway Ventilation (Expiratory Positive Airway Pressure, Inspiratory Positive Airway Pressure, Non-positive Pressure, Mean Airway Pressure)

#### Selected hyperparameters

*GridSearchCV* from the *scikit-learn* [29] library selected hyperparameters for all classifiers though some variables, increments, and boundaries were manually fixed to constrain the search space. For the ridge regression classifier, it chose an inverse regularization strength (C) of 0.01 on the LIBLINEAR solver. For the multi-layer perceptron classifier with a single hidden layer of size 10, it chose a rectified linear unit (ReLU) function for the hidden layer, the Adam solver for weight optimization, and an alpha (L2 penalty) of 1.0. For the random forest classifier, it chose to use 120 trees, a maximum depth of 12 for each tree, a minimum of 5 samples per leaf node, and a maximum of 5 features per split. For the XGBoost classifier, it chose to use 180 estimators, a maximum depth of 2, a learning rate of 0.1, and a gamma (minimum loss reduction for each partition) of 0.

#### Classifier performance

**Fig. 2:**
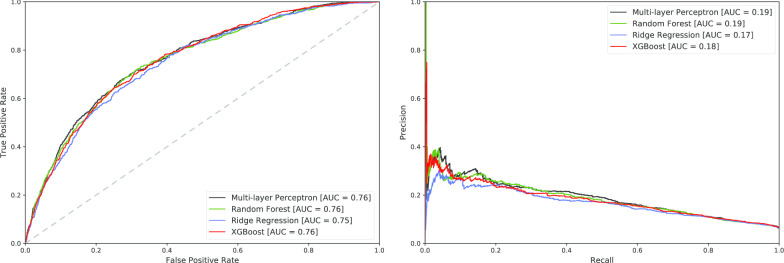
Receiver operating characteristic (ROC) and precision-recall curves of the testing set

Performance was similar across all four classifiers. The multi-layer perceptron had the highest AUC (0.76) and highest area under the precision-recall curve (0.19). With the threshold determined by Youden’s J statistic, the multi-layer perceptron achieved a precision of 0.1424 and F_1_-score of 0.2351. As shown in Fig. 2 and Table 4, ridge regression performed similarly in all metrics, especially when recall is high and clinically meaningful. Given comparable performance, regression classifiers may be preferable for clinical interpretation as regression coefficients are generated for each feature.

Table 4 demonstrates a trade-off in which recall is preferred over precision. This prioritization is desirable for our use case as a high detection rate (recall) for patients at risk of hyperchloremia is useful, whereas false positive alerts (precision) are generally tolerable.

**Table 4:** Precision, Recall, and F$_{1}$-scores of the testing set using thresholds set by the maximal Youden’s J statistic

Model	Precision	Recall	F$_{1}$-score
Multi-layer perceptron	0.1424	0.6741	0.2351
Random forest	0.1423	0.6741	0.2350
Ridge regression	0.1431	0.6169	0.2323
XGBoost	0.1405	0.6550	0.2314

These results can also be represented using the number needed to alert (NNA = 1/precision) [30]. In the case of a baseline “model” in which all patients are flagged for next-day hyperchloremia, the precision would equal the prevalence (0.06), which then translates to a NNA of 17. In comparison, based on a precision of 0.14, our models perform considerably better with a NNA of 7. While an even lower NNA may be needed to justify high-risk interventions with stringent cost-benefit considerations, this improvement could provide reasonable justification for low-risk, low-cost chloride-reducing adjustments. For example, a clinician could switch to low-chloride fluids and/or administer loop diuretics sooner—generally safe and predictable chloride-reducing actions.

#### Feature analysis

Ridge regression coefficients with magnitude greater than 0.2 are reported in Table 5. As we can see, maximum serum chloride on day 1 had the largest influence. This is understandable as one would expect that serum chloride on day 1 is a strong predictor of serum chloride on day 2. Chloride load also had a relatively large positive coefficient and follows a similar line of reasoning. Interestingly, paralysis, mean airway pressure ventilation, female gender, Asian ethnicity, and increased age were each associated with greater likelihoods of developing hyperchloremia.

**Table 5:** Ridge regression coefficients (magnitude > 0.2)

Feature	Coefficient
Max. chloride	0.6332
Chloride load	0.4064
Paralysis (C)	0.3353
Mean airway pressure (I)	0.2961
Female	0.2665
Max. sodium	0.2621
Asian	0.2609
Age	0.2210
Max. potassium	− 0.2537
Min. bicarbonate	− 0.2705
Renal failure (C)	− 0.2946
Complicated diabetes (C)	− 0.3409
Non-positive pressure (I)	− 0.4681

*Binary features: (C)=comorbidity, (I)=intervention*

#### Error analysis

We selected 10 ICU stays that were incorrectly classified by ridge regression to investigate by hand. Specifically, we identified from the training set the five false positives with the highest predicted probabilities of hyperchloremia on day 2 and the five false negatives with the lowest predicted probabilities.

The five false positives were each predicted to develop hyperchloremia with greater than 98% probability. Two of the five cases were in fact on the cusp of our cutoff for hyperchloremia on day 1, with maximum serum chloride measurements of 109 mEq/L, but did not develop hyperchloremia ($\ge {110}$ mEq/L) on day 2. Another two also had high measurements on day 1, 108 mEq/L and 107 mEq/L. All five patients received a significant amount of chloride load and net fluid input, averaging more than 1500 mEq and 17 L respectively over the first day. Given the large coefficients for serum chloride and chloride load, these borderline measurements and large inputs most likely account for the misclassifications. Addressing these discrepancies would likely require the addition of more discerning features or a change to our definition of hyperchloremia. Interestingly, all five patients had abnormally low minimum serum bicarbonate, ranging from 20 mEq/L to 6 mEq/L, and all five were on mean airway pressure ventilation.

In contrast, the five false negatives were each predicted to develop hyperchloremia with lower than 10% probability. All five cases had relatively low serum chloride on day 1, each with maximum serum chloride of 102 mEq/L or lower. Other features (e.g. chloride load, fluid balance) are quite unremarkable for these patients, with no notable trends or large values. Further examination of these patient records revealed that one patient began receiving a high chloride load on day 2 before developing hyperchloremia on the same day. Records for another patient suggested a measurement error or very sudden and sharp hyperchloremia, demonstrating consistently low serum chloride measurements ($\le {103}$ mEq/L) throughout day 2 with the exception of one measurement that was not precipitated by chloride administration and was well above our cutoff (121 mEq/L). The other three patients exhibited gradual variations in serum chloride that briefly and slightly crossed our cutoff on day 2 without the administration of significant amounts of chloride. This analysis demonstrates the limitations of assumptions made during model development and the consequences of noisy clinical data.

The confusion matrix for our ridge regression classifier’s training set predictions is reported in Table 6, which shows that this model ultimately predicted 5,460 false positives among 21,968 patients that did not develop hyperchloremia.

**Table 6:** Confusion matrix for the ridge regression training set

	Predicted non-hyperchloremic	Predicted hyperchloremic
True non-hyperchloremic	16,508	5460
True hyperchloremic	464	899

Regression analysis of this false positive subgroup revealed increased odds of ICU mortality, new AKI by day 7, and MODS on day 7 that were statistically significant when compared to the true negative subgroup (Table 7).

**Table 7:** Comparing false positive patient outcomes to true negative patient outcomes under the ridge regression model

Outcome	$\chi ^2$ *p* value	Odds ratio [95% CI]
ICU mortality	$< 0.001$	2.218 [1.984, 2.481]
New AKI by Day 7	$< 0.001$	1.570 [1.473, 1.673]
MODS on Day 7	$< 0.001$	1.741 [1.495, 2.027]

## Discussion

After adjusting for confounders, we observe that elevated serum chloride and increased chloride load are both associated with higher mortality rates in a general ICU population. Patients with increased measures of serum chloride also demonstrate increased adjusted odds of new AKI by day 7 and MODS on day 7. These findings are consistent with the existing literature on ICU subpopulations, which have reported increased mortality with high chloride levels as well as reductions in kidney injury with low-chloride treatment strategies [2, 3, 6]. Our study corroborates these findings in a broad ICU population of 48,074 patients, further generalizing what is understood about the potential effects of elevated chloride.

Using information available from the first day of ICU stays, we constructed a set of 34 features and trained classifier models to predict the occurrence of hyperchloremia on the second day. As far as we know, this is the first implementation of hyperchloremia prediction using machine learning models. Performance was similar across the multi-layer perceptron, random forest, ridge regression, and XGBoost models, with typical AUCs of approximately 0.76 and NNAs of approximately 7.

As we grow our understanding of how chloride load and hyperchloremia may affect morbidity and mortality, these prediction classifiers produce alerts that are clinically actionable. Clinicians can provide targeted care to high-risk patients by modifying chloride administration and elimination strategies, via the use of low-chloride fluids, diuretics, or other interventions. Implementing these strategies in *all* ICU patients could be cost-prohibitive and cumbersome, and thus identifying high-risk patients offers an opportunity for a more directed and efficient approach. With the relatively low number needed to alert seen in our models, there is potential for significant cost reductions if such changes are implemented.

While false positive alerts may be technically incorrect when evaluating classifier performance, we do observe higher rates of ICU mortality, new AKI by day 7, and MODS on day 7 in this group when compared to the true negative cohort. Given their higher risk of poor outcomes, this subgroup of false positive alerts may also benefit from closer consideration and modified treatment strategies.

### Limitations and future work

Since we have taken a focused approach on aggregated data from the first day of ICU care, there is much potential in expanding our features to include longitudinal trends in patient data and make continuous, rolling predictions. Additionally, MIMIC-III contains a considerable amount of information that we have yet to explore—its clinical notes, for example, contain much textual data that was not considered in this study. More complex and subtle feature engineering could improve performance and discern interesting subgroups of patients, including those that may not fit well under our current model.

Further work should also evaluate other ICU cohorts including those of specialized ICUs (e.g. pediatric ICUs) so that comparisons and generalizations can be made across different datasets while accounting for unique clinical considerations and feature constraints.

Lastly, our findings are ultimately drawn from correlations, and continued research should probe for causal links and evaluate the efficacy of interventions for improving outcomes. Interventions should also be considered for false positive patients, who exhibit patterns of poor outcomes and thus could also benefit from closer observation. Evaluation of such interventions can then lead to evidence-based changes in clinical care.

## Conclusions

Our regression analysis has shown hyperchloremia during the acute phase of critical illness to be independently associated with increased ICU mortality, new AKI by day 7, and MODS on day 7 in a general ICU population. In addition, we demonstrate an independent association between chloride load in IV fluids and increased ICU mortality. These findings warrant closer attention to chloride management in critically ill patients.

Our supervised learning classifiers are able to predict next-day hyperchloremia at clinically-actionable performance levels using features from the first days of patient ICU stays. These classifiers yield a number needed to alert of 7 while maintaining acceptable levels of recall—a helpful rate considering the low prevalence of new hyperchloremia in the ICU. Furthermore, error analysis reveals a familiar trend of increased morbidity and mortality among false positive predictions.

With the potential to help prevent hyperchloremia, these predictive models are stepping stones towards supporting clinicians as we optimize clinical care and improve patient outcomes. There is also much potential in future work, which should validate these models in additional ICU cohorts, broaden the scope of features used, and evaluate potential interventions for at-risk patients to translate this progress into clinical action.

## Data Availability

The MIMIC-III dataset is freely accessible at https://mimic.physionet.org/. Notebooks containing our code are available at https://github.com/chloride-management/chloride-management/.

## Abbreviations

AKI: Acute Kidney Injury
AUC: Area Under the Receiver Operating Characteristic Curve
EHR: Electronic Health Record
ICU: Intensive Care Unit
IV: Intravenous
MIMIC-III: Medical Information Mart for Intensive Care III
MODS: Multiple Organ Dysfunction Syndrome
NNA: Number Needed to Alert
ROC: Receiver Operating Characteristic
SOFA: Sequential Organ Failure Assessment
